# Exploring the Limits of Polyhydroxyalkanoate Production by Municipal Activated Sludge

**DOI:** 10.1021/acs.est.2c03043

**Published:** 2022-07-28

**Authors:** Ruizhe Pei, Ángel Estévez-Alonso, Laura Ortiz-Seco, Mark C. M. van Loosdrecht, Robbert Kleerebezem, Alan Werker

**Affiliations:** † Department of Biotechnology, Delft University of Technology, Van der Maasweg 9, 2629 HZ Delft, The Netherlands; ‡ Wetsus, European Centre of Excellence for Sustainable Water Technology, Oostergoweg 9, 8911 MA Leeuwarden, The Netherlands

**Keywords:** resource recovery, municipal wastewater treatment, activated sludge, biopolymers, polyhydroxyalkanoate (PHA)

## Abstract

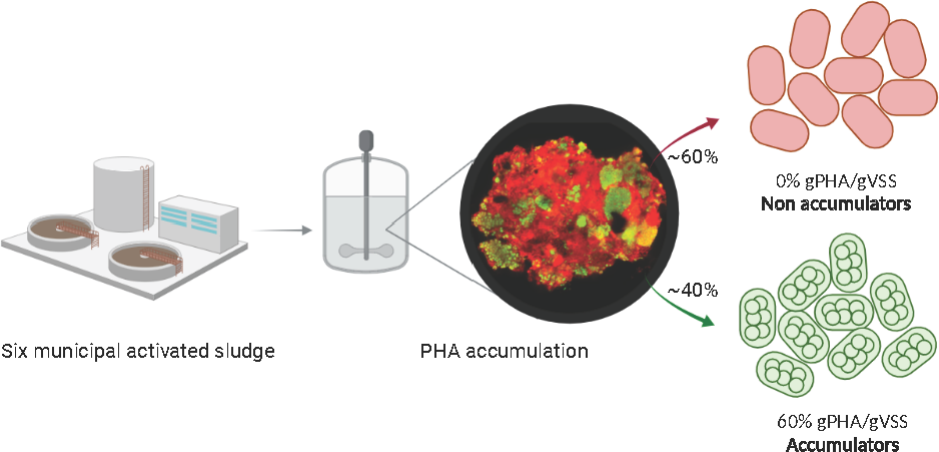

Municipal activated sludge can be used for polyhydroxyalkanoate (PHA) production, when supplied with volatile fatty acids. In this work, standardized PHA accumulation assays were performed with different activated sludge to determine (1) the maximum biomass PHA content, (2) the degree of enrichment (or volume-to-volume ratio of PHA-accumulating bacteria with respect to the total biomass), and (3) the average PHA content in the PHA-storing biomass fraction. The maximum attained biomass PHA content with different activated sludge ranged from 0.18 to 0.42 gPHA/gVSS, and the degree of enrichment ranged from 0.16 to 0.51 volume/volume. The average PHA content within the PHA-accumulating biomass fraction was relatively constant and independent of activated sludge source, with an average value of 0.58 ± 0.07 gPHA/gVSS. The degree of enrichment for PHA-accumulating bacteria was identified as the key factor to maximize PHA content when municipal activated sludge is directly used for PHA accumulation. Future optimization should focus on obtaining a higher degree of enrichment of PHA-accumulating biomass, either through selection during wastewater treatment or by selective growth during PHA accumulation. A PHA content in the order of 0.6 g PHA/g VSS is a realistic target to be achieved when using municipal activated sludge for PHA production.

## Abstract 2

Activated sludge has shown different biomass PHA content. In this work, differences in PHA content were related to the abundance of PHA-storing microorganisms in the activated sludge, and subsequently the maximum biomass PHA content was derived.

## 1 Introduction

Municipal wastewater treatment plants (WWTP) rely on the use of complex microbial communities to efficiently treat and robustly remove and/or recover carbon, nitrogen, phosphorus, as well as other selected forms of contamination from wastewater.^1,2^ Different process configurations have evolved over the last century to efficiently remove these contaminants, involving nitrification–denitrification and enhanced biological phosphorus removal.^3,4^ Different bioprocess configurations will create environmental pressures to select for different microbial communities. These microbial communities directly relate to process functional performance.^5−7^ For instance, biological phosphorus removal selects for polyphosphate-accumulating bacteria and nitrification–denitrification selects for denitrifying heterotrophic bacteria. Selective pressures can be due to alternation of anaerobic, anoxic, and aerobic process stages where different types of microorganisms may use different kinds of carbon sources, electron donors, electron acceptors, and/or energy sources present in the influent wastewater. Because of these alternating conditions, WWTPs often impose very dynamic environments for microorganisms and often harbor an enormous diversity of microorganisms.

Dynamic environments also tend to enrich for microorganisms that are able to store intracellular compounds as carbon or energy reservoirs. Such storage allows for effective growth in dynamic environments. For example, polyphosphate, glycogen, and/or polyhydroxyalkanoates (PHA) may be accumulated by certain species of bacteria.^8,9^ Polyphosphate and glycogen-storing microorganisms are capable of using intracellular polyphosphate and glycogen pools as energy sources to take up and store an external substrate in the form of PHA in the absence of an electron acceptor. PHA is normally produced intracellularly as energy and carbon storage polymers to deal with the alternating presence and absence of carbon source or electron acceptor.^8,10,11^ Most aerobic and anoxic bacteria can store PHA as carbon and energy reservoirs when organic carbon is available but other nutrients for microbial growth are (temporarily) missing. The stored PHA can be used for microbial growth when no external organic carbon source is available but all other intra- or extracellular growth factors are sufficiently present.

PHAs stored in biomass can be recovered as biodegradable polymers with thermoplastic and mechanical properties of interest for bioplastic formulations and industrial applications.^12,13^ The surplus activated sludge produced in municipal WWTPs can be a biomass resource to produce PHA, if a suitable feedstock rich in volatile fatty acids is supplied to this biomass in a PHA accumulation process.^14,15^ The direct use of waste activated sludge to produce PHA, without further enrichment, has been widely studied in recent years at lab and pilot scales.^12,13^ However, the reported maximum achieved PHA content with waste activated sludge has been lower on average than those obtained when a PHA-producing enrichment culture is used, 0.4–0.6 gPHA/gVSS compared to 0.4–0.9 gPHA/gVSS. One reason for lower observed PHA content is anticipated because of an expected lower fraction of PHA-accumulating bacteria in municipal activated sludge. Still, the achieved PHA content is greater than 0.4 gPHA/gVSS, which has been reported to be the minimum threshold for making an economically viable PHA production process.^15^

Determination of the biomass (average) PHA content is most commonly reported on a mass basis,^12,16^ as grams of PHA with respect to the total biomass in the sample represented by volatile suspended solids (VSS). With this metric, it is not possible to distinguish the contribution of individual populations to the total PHA production. In a recent work, a new staining method with microscopy and image analysis was developed and applied to differentiate and quantify between the PHA-accumulating and non-PHA-accumulating biomass fractions in activated sludge.^17,18^ With this tool, the degree of enrichment for the PHA-accumulating biomass fraction was estimated and directed toward understanding for variations in PHA production processes due to combined factors of amount of polymer stored and fraction of the biomass actively storing polymers. This created an opportunity to explore intrinsic characteristics of microbial cultures to be used for PHA production. With the combined evaluation of maximum biomass PHA content and the biomass degree of enrichment, the biomass PHA content in the PHA-accumulating biomass fraction was estimated.

The aim of this work was to critically assess the PHA accumulation potential of activated sludge from different municipal WWTPs. The goal was to determine the degree of enrichment for PHA-accumulating bacteria and to reveal limits for directly using surplus activated sludge as a biomass source for industrial scale PHA production. PHA accumulation potential assays for activated sludge sourced from a set of six different municipal WWTPs were assessed in combination with the biomass degree of enrichment for PHA-accumulating bacteria. Results and insights from the established standardized PHA accumulation methods together with selective complementary staining with confocal microscopy image analyses are reported and discussed herein.

## 2 Materials and Methods

### 2.1 Sludge Source and Feedstock

Grab samples of activated sludge from six different municipal WWTP were used for standardized PHA accumulation assays (Table 1). The set of WWTPs were selected based on process configuration, either nitrification and denitrification with chemical phosphorus removal (AO) or biological phosphorus removal (A^2^O). Some WWTPs had also been evaluated in a previous study, and this allowed for direct comparison of results.^15^ Mixed liquor samples (5 L) were obtained from the main aerobic process and were settled for 30–60 min. The supernatant was decanted, and settled activated sludge was delivered on the same day to Wetsus (Leeuwarden, The Netherlands) by courier. Samples were stored at 4 °C pending assays. The experimental period was between March and June 2021.

**Table 1:** Selected Municipal WWTPs^a^

WWTP	country	capacity (kPE)	process	P-removal	primary settling
Bath	NL	536	AO	chemical	yes
Leeuwarden	NL	250	AO	chemical	no
Beverwijk	NL	351	AO	chemical	yes
Almere	NL	329	A^2^O	biological	no
Dordrecht	NL	310	A^2^O	biological	no
Winsum	NL	23	AO	chemical	no

a AO, anoxic-aerobic; A^2^O, anaerobic-anoxic-aerobic. More information about the selected WWTPs can be found in the WAVES dashboard (https://live-waves.databank.nl/).

The accumulation feedstock, with nutrient ratio 100:1:0.05 (COD:N:P by weight), was prepared with tap water as follows: 50 g/L acetic acid, 1.91 g/L NH_4_Cl, and 109.6 mg/L KH_2_PO_4_. The feedstock pH was adjusted to pH 4.5 with KOH pellets.

### 2.2 PHA Accumulation Assays

PHA accumulation assays were performed over 48 h in a double-jacketed glass bioreactor (1 L working volume) at 25 ± 0.1 °C. Agitation at 150 rpm was accomplished by standard three-bladed turbine (R60, CAT Scientific, Germany). pH was monitored but not controlled and ranged from 7.5 to 9.0. The airflow rate was fixed at 1 L/min (MV-302, Bronkhorst, Germany). Dissolved oxygen and pH probes (COS81D and CPS11D, Endress & Hausser, The Netherlands) were coupled to a 4-channel transmitter (Liquiline CM444, Endress & Hausser, The Netherlands), and measurements were logged every 10 s. Probes were calibrated according to manufacturer instructions for each assay. Substrate dosing diaphragm pumps (Stepdos 10, KNF, The Netherlands) were actuated by PLC (Logo! Eight and Logo! TDE, Siemens, Germany).

The standard PHA accumulation assay was performed to evaluate a PHA accumulation potential for the different municipal activated sludge sources. For each assay, gravity-thickened activated sludge samples were diluted with tap water to nominally 2–3 gVSS/L, and allylthiourea (50 mg/L) was added directly to the reactor to inhibit nitrification. The mixed liquor was brought to 25 °C and conditioned with constant aeration overnight to establish a baseline of endogenous microbial activity in all cases. Subsequently, an automated acclimation comprising three feast and famine cycles was applied as previously reported.^19^ Feast conditions were generated with a pulse input to reach a maximum substrate level of 150 mgCOD/L, and the duration of the feast was monitored by changes in respiration based on dissolved oxygen trends. The famine period was dynamically adjusted to be three times longer than each respective feast time. The duration of each feast/famine cycle was dependent on the activated sludge used, and it ranged from 1 to 3 h. Trends in respiration were used to estimate the oxygen mass transfer coefficient (k_L_a). After the third famine period, the accumulation assay was started automatically. Accumulation was driven with the same feast influent pulses and control logic, but now without any famine period between pulses. Pulse inputs were controlled from online monitoring of dissolved oxygen according to Valentino et al.^20^

### 2.3 Staining and Microscopy Image Analysis

Mixed liquor grab samples were taken at selected time points during the PHA accumulation assays. Samples were fixed with formaldehyde to a final concentration of 3.7% and preserved in a 1:1 ratio mixed with 1X phosphate-buffered saline and pure ethanol before storing at −20 °C. The staining of PHA and non-PHA biomass was performed with BODIPY 493/503 (BODIPY) (Thermo Fisher Scientific, MA, United States) in combination with Sypro Red (Thermo Fisher Scientific, MA, United States), according to Pei et al.^17^ Fixed sample aliquots of 5 μL were loaded in reaction wells (10 per glass slide). Reaction wells were further provided with 0.5 μL of BODIPY at 2 ng/μL and 0.5 μL of 100 times diluted Sypro Red. The glass slides were dried at 46 °C. Residual dye was rinsed from the dried slide with Milli-Q water, and slides were then dried again with compressed air before mounting with Vectashield HardSet Antifade Mounting Medium H-1400-10 and sealing.

The fixed and stained samples were evaluated by a Confocal Laser Scanning Microscope LSM 880 (Carl Zeiss, Germany) with Plan-Apochromat 63×/1.4 Oil DIC objectives (Carl Zeiss, Germany). Methods of image capture were as described in Pei et al.^17^ Each reaction well was first surveyed to get an overall impression. Then, images from 10 randomly selected fields of view containing floc structures were acquired. For each field of view, BODIPY and Sypro Red were excited with an argon laser (488 nm) and a DPSS 561-10 laser (561 nm), respectively. Overlay images were captured into separate image channels. For each channel, 16 scans were averaged and stored at 16-bit depth. Conditions of laser power and gain were conserved in the set of 10 images (2 channels per image), and imaging conditions were otherwise kept similar from well-to-well.

Images were evaluated in Fiji ImageJ (ImageJ2, Ver. 1.52P). For each set of images, brightness was maximized, without overexposing for individual pixels, and the cutoff for background threshold intensity level was established by visual inspection. Total pixel counts representing PHA and protein (non-PHA biomass) volumes in the plane of focus for activated sludge flocs in each field of view were measured.

For each field of view, the relative area ratio for PHA to non-PHA biomass ratio (v/v) was calculated:
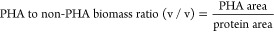
1

The average ratio from 10 fields of view represented the estimated ratio of PHA to non-PHA biomass (v/v) for each well. The activated sludge degree of enrichment was defined as the average PHA to non-PHA biomass ratio (v/v) that was reached by the end of the accumulation assay.

### 2.4 Analytical Methods

PHA accumulation assay outcomes were assessed with online logged measurements (DO, pH, and temperature) and with solid and liquid analyses from grab samples of mixed liquor at selected time points in replicates of 3 × 15 mL. Suspended solids were separated by centrifugation (3250 rcf and 4 °C for 20 min). The supernatant was stored at −20 °C pending liquid analyses after membrane filtration (0.45 μm pore size filters). The suspended solids pellet dry and ash weights were estimated based on standard methods for solids analyses.^21^ Total and volatile suspended solids (TSS and VSS) concentrations were then estimated with respect to the 15 mL sample volume. Acetic acid concentration was determined by ultrahigh pressure liquid chromatography, and ammonium, nitrite, nitrate, and phosphate concentrations were determined by ion chromatography, as previously reported.^22^

One of the 15 mL aliquots was used for PHA determination. The liquid volume was directly acidified to pH 2 with 37% HCl. The acidified suspended solids were thoroughly mixed for 5 min and centrifuged (3250 rcf and 4 °C for 20 min). The harvested pellet was dried at 105 °C overnight and ground. Average biomass PHA content was estimated by thermogravimetric analysis (TGA) as previously reported.^23^

### 2.5 Data Analysis

All measured parameters were corrected for effects of sample withdrawal and feedstock addition from liquid and mass balance considerations.^24^ The biomass PHA content that was measured as a function of time was expressed as mass fraction of the volatile suspended solids (gPHA/gVSS). Active biomass (X_a_) was estimated as VSS minus PHA mass. Active biomass was assumed to be represented as CH_1.8_O_0.5_N_0.2_.^25^ The trend for accumulated biomass PHA content was represented by least-squares regression to the empirical function as in Bengtsson et al.:^15^
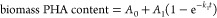
2where *A*_0_ is the theoretical initial PHA content, *A*_1_ the theoretical maximum PHA content, and *k*_1_ a constant that enabled estimation of rates as a function of time and comparison of performance for different activated sludge sources. The accumulation time constant τ (τ = 1/*k*_1_ (h)) represented process first-order kinetics in reaching a maximum level of PHA content. Initial and average specific production/consumption rates and PHA yields on substrate were estimated for process times of 0.2τ and 3τ, respectively. The times of 0.2τ and 3τ were when biomass reached 18% and 95% of maximum PHA content, respectively. In assays where 3τ was longer than the accumulation assay period, yields and rates are reported for the last sampling time instead. The average PHA yields on the substrate were calculated on a COD-basis assuming poly(3-hydroxybutyrate) (1.67 gCOD/gPHB) produced on acetate (1.07 gCOD/gHAc) added. Average specific production and consumption rates were calculated from the cumulative amounts of acetic acid, PHA, biomass, and oxygen consumed with respect to estimated active biomass levels (gCOD/gX_a_/h).

The trends of PHA to non-PHA biomass ratio (v/v) as a function of time could also be similarly fitted by least-squares regression analysis to the first-order rate equation:
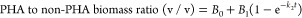
3where *B*_0_ is the theoretical initial ratio, *B*_1_ the theoretical final ratio, and *k*_2_ a constant that characterized the development of PHA distribution in the biomass as the PHA to non-PHA biomass ratio (v/v) for the different activated sludge sources.

The average PHA content for the fraction of the PHA-accumulating biomass in the activated sludge was determined by
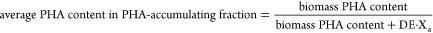
4where DE is the degree of enrichment defined. DE was estimated by the level of PHA to non-PHA biomass ratio (v/v) that evolved by the end of the accumulation assay.

## 3 Results

The outcomes for the standardized PHA accumulation assays from the six sources of municipal activated sludge are summarized in Table 2. From these assays, the degree of enrichment for PHA-accumulating biomass fraction for the activated sludge was determined, and results are given in Table 3.

**Table 2:** Summary of the PHA Accumulation Assay Results^a^

WWTP	max. PHA content (gPHA/gVSS)	τ (h)	*q*_HAc_^0.2τ^ (gCOD/gX/h)	*Y*_PHA,HAc_^0.2τ^ (gCOD/gCOD)	*q*_HAc_^3τ^ (gCOD/gX/h)	*Y*_PHA,HAc_^3τ^ (gCOD/gCOD)
Bath	0.37	5	246	0.46	137	0.47
Leeuwarden	0.30	12	111	0.45	64	0.25
Beverwijk	0.42	17	142	0.43	108	0.24
Almere	0.18	7	84	0.46	47	0.31
Dordrecht	0.32	17	90	0.32	81	0.21
Winsum	0.23	10	141	0.17	51	0.16

a *q*_HAc_ stands for acetate biomass uptake rate, and *Y*_PHA,HAc_ stands for average yield of PHA produced on acetate feed. 0.2τ and 3τ were when biomass reached 20% and 95% of maximum PHA content, respectively.

**Table 3:** Degree of Enrichment and PHA Content in the PHA-Accumulating Biomass Fraction Accumulation Assays (*X*_PHA_)

WWTP	PHA content (gPHA/gVSS)	DE (v/v)	PHA content in *X*_PHA_ (gPHA/gVSS)
Bath	0.37	0.51	0.54
Leeuwarden	0.30	0.36	0.55
Beverwijk	0.42	0.42	0.61
Almere	0.18	0.31	0.46
Dordrecht	0.32	0.26	0.67
Winsum	0.23	0.16	0.66

### 3.1 PHA Accumulation Performance

**Figure 1:**
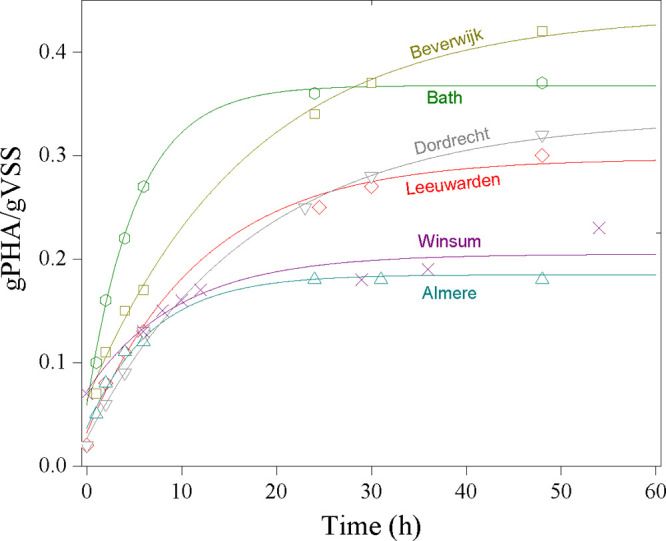
PHA accumulation trends for all the assayed WWTPs. Symbols represent the measured values, and the trend lines are from eq 2.

In general, PHA levels increased over the course of the accumulation assay and asymptotically approached a maximum level of biomass PHA content. The measured plateau PHA content was typically reached between 24 and 60 h in most cases, and levels remained constant for the remaining duration of the assays (Figure 1). The maximum biomass PHA content ranged from 0.18 to 0.42 gPHA/gVSS with an average value of 0.30 ± 0.08 gPHA/gVSS. When clustered by the type of WWTP configuration, AO WWTPs showed an average biomass PHA content of 0.33 ± 0.07 gPHA/gVSS (*n* = 4) while A^2^O WWTPs had an average of 0.25 gPHA/gVSS (*n* = 2). When clustered by the presence and absence of primary treatment, WWTPs with primary treatment showed an average biomass PHA content of 0.40 gPHA/gVSS (*n* = 2) while WWTPs without primary treatment had an average of 0.26 ± 0.07 gPHA/gVSS (*n* = 4). Initial PHA yields on the substrate were in the range of 0.4–0.7 gCOD_PHA_/gCOD_HAc_. However, the PHA yield per amount of acetate fed decreased significantly as the maximum PHA content was attained. The PHA yield on the substrate diminished to levels that were below 0.10 gCOD_PHA_/gCOD_HAc_. Consequently, by the end of the accumulation assay, the average PHA yields were not greater than 0.30 gCOD_PHA_/gCOD_HAc_. This decrease indicated that there was essentially no net PHA production in the latter stages of the accumulation assays.

**Figure 2:**
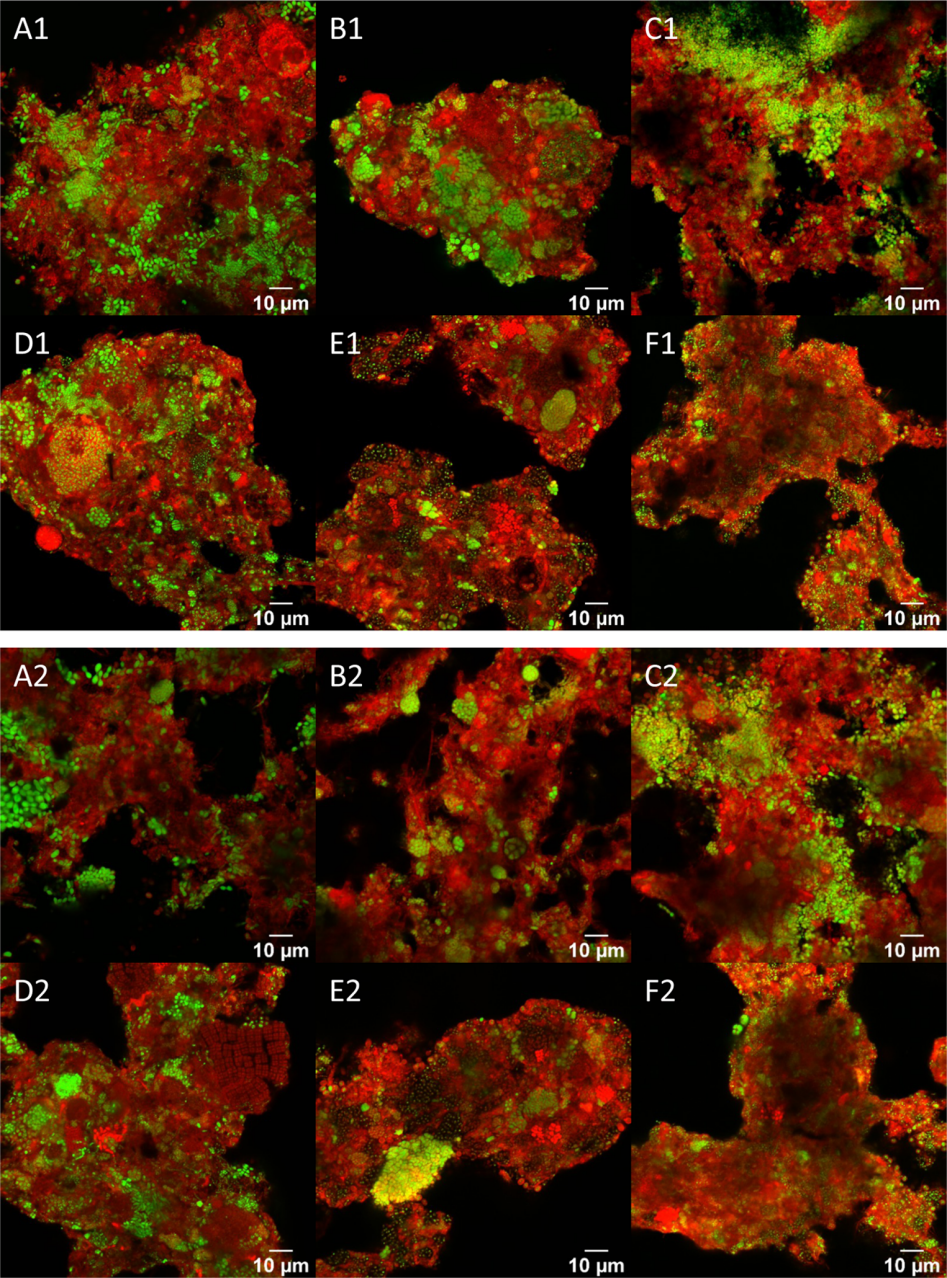
Stained PHA granules (green) and non-PHA biomass (red) after 48 h of accumulation from WWTP of Bath (A), Leeuwarden (B), Bevewijk (C), Almere (D), Dordrecht (E), and Winsum (F) at different fields of view.

Some active biomass growth was observed toward the end of the accumulations, but not at the beginning. Average active biomass yields on substrate were low at the beginning of the accumulation, <0.05 gCOD_X_/gCOD_HAc_, but increased over time to levels in the range of 0.01–0.24 gCOD_X_/gCOD_HAc_. This development supports that polymer storage was more significant than active biomass growth during the initial stages of the accumulation assay. Beverwijk WWTP was a noted exception. In this case, active biomass growth was observed directly from the start of accumulation. Despite the observed increasing active biomass concentrations in the latter part of assays, biomass PHA content was found to continue to increase slowly (Figure 2). COD mass balances that were estimated from measured and estimated values could not be closed. Initially (0.2τ) and at the later stages (3τ hours), only 70 ± 21% and 69 ± 23% of acetate as COD removed, respectively, could be accounted for as the sum of PHA and biomass produced plus oxygen consumed.

### 3.2 PHA Distribution in Activated Sludge Flocs

Images with the selectively stained components revealed that biomass in flocs dominated, and levels of free living bacteria were considered to be relatively low. Coverage of PHA in the flocs increased on average during all the assays. However, by the end of the assays, still just a fraction of the biomass exhibited accumulated PHA, as shown in Figure 2. The observed morphology of the PHA-accumulating bacteria was diverse for different activated sludge, including rod shape, filaments, and cocci. Image resolution was sufficient in some cases to observe a range of 1–8 individual intracellular PHA granules per cell.

The fraction of PHA-storing biomass was observed to be heterogeneously distributed within and between flocs. PHA storing activity tended to develop as aggregated clusters within individual flocs. Thus, selection for the PHA-storing phenotype was generally not considered to be uniformly distributed within the municipal activated sludge. One exception was Winsum WWTP. In this case, PHA-accumulating bacteria were notably spread across observed floc volumes.

### 3.3 Degree of Enrichment and Average PHA Content in the PHA-Accumulating Fraction

**Figure 3:**
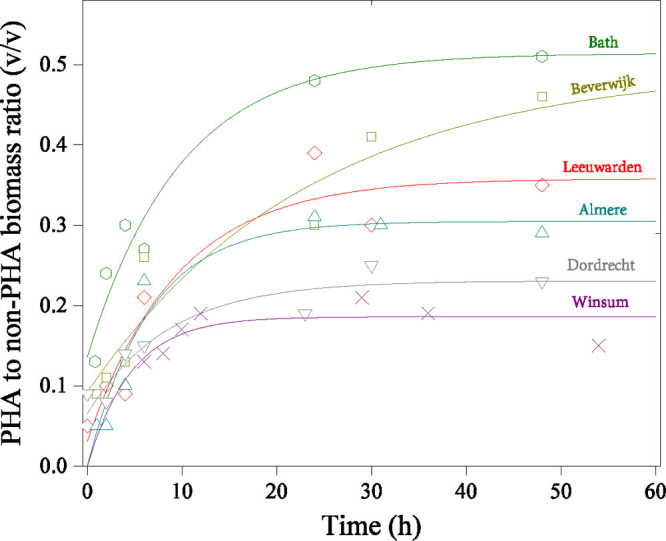
Development of PHA to non-PHA biomass ratio (v/v) during PHA accumulation assays. Symbols are the measured values from image analyses, and the trend lines are from eq 3.

Figure 2 depicts typical observations indicating how not all the biomass was found not be actively engaged in PHA storage. The trend of PHA to non-PHA biomass average ratios (v/v) followed by analogy to trends of PHA content according to eq 3, as observed in Figure 3. The average PHA to non-PHA biomass ratio increased asymptotically toward a plateau value by 48 h. WWTP Beverwijk was again the exception. This activated sludge exhibited a progressively increasing trend toward higher levels. These concurrent trends of average biomass PHA content and degree of enrichment from six municipal activated sludge sources replicated the experience previously observed by Pei et al.^17,18^ with activated sludge from Bath WWTP. A degree of enrichment of 1 would be indicative of a biomass with 100% selection of the PHA-storing phenotype.^18^ In the present work, with levels of less than 0.51 for degree of enrichment, not more than about half of the biomass was active in PHA storage during the assays. The estimated levels of degree of enrichment could not be readily coupled to be systematically higher or lower for either biological phosphorus removal or nitrification and denitrification WWTP process configurations. A^2^O WWTPs (Almere and Dordrecth) showed degrees of enrichment in the range of 0.26–0.31 while AO WWTPs (Bath, Leeuwarden, Beverwijk, and Winsum) ranged from 0.16 to 0.51.

The principal assay outcomes were the degree of enrichment and the biomass PHA content. These data enabled estimating the average PHA content for the PHA-accumulating biomass fraction (eq 4). A consistently high average level of PHA storage was estimated for all activated sludge sources. The average PHA content in the PHA-accumulating biomass fraction was 0.58 ± 0.07 gPHA/gVSS. At a PHA content of 0.67 gPHA/gVSS, as observed in Dordrecht WWTP, the polymer to active biomass mass ratio is 2. Thus, the PHA-accumulating bacteria from these municipal activated sludge sources exhibited similar capacities to reach up to double their organic mass as polymer.

## 4 Discussion

### 4.1 Municipal Activated Sludge Accumulates up to 0.58 gPHA/gVSS

Microbial community-based PHA production has been widely studied over the past 20 years. However, the direct use of municipal waste activated sludge, without further enrichment, has received less research attention.^12,13,26^ In the present work, different municipal WWTPs have exhibited different PHA accumulation potentials ranging from 0.18 to 0.42 gPHA/gVSS. These results are in line with previous experiences of PHA production with municipal activated sludge fed with synthetic feedstocks and fermented waste streams.^15,27^ These levels are still much lower than the maximum levels that have been obtained with highly enriched cultures, where PHA content of up to 0.9 gPHA/gVSS has been attained with synthetic feedstocks.^28^ Notwithstanding, the range of PHA content reached for enrichment cultures produced on fermented wastewater has been in the range from 0.6 to 0.8 gPHA/gVSS.^13^ From the present investigation, it is confirmed that outcomes for direct accumulation using municipal waste activated sludge are challenged by the presence of non-PHA-storing bacteria.

PHA accumulation patterns and PHA granule morphology were found to be diverse among the different activated sludge sources used, suggesting a high diversity of PHA-accumulating microorganisms within and between different WWTPs. Even though these different microorganisms may have different respective maximum PHA contents, it was surprising to observe that, on average, the PHA content in the PHA-accumulating biomass fraction was observed to be in the range of 0.5–0.7 gPHA/gVSS. This observation also suggests that it is realistic to attain PHA content with municipal activated sludge of up to about 0.6 gPHA/gVSS. This level is significantly higher than those generally observed and historically expected with direct accumulation for waste activated sludge, and it is in line with the maximum values ever reported for the direct use of municipal activated sludge for PHA production.^27,29−31^ If the upper limits (0.6–0.7 gPHA/gVSS) can be consistently obtained with municipal activated sludge, it would enable much broader generic potential to source biomass for direct PHA production. Wider generic availability of PHA-producing biomass would facilitate supporting PHA polymer value chains and, thereby, growth of biopolymers and chemical biobased industrial sectors.

Why the PHA-storing phenotype in municipal activated sludge accumulates an average of 0.6 gPHA/gVSS and not a greater amount could not be evaluated as part of this work. A similar line of discussion is found in the literature for enrichment cultures. While highly enriched cultures have shown biomass PHA content up to 0.9 gPHA/gVSS, not all enrichment cultures have resulted in such extraordinarily high PHA levels, and PHA content in the range from 0.5 to 0.8 is commonly reported.^12,13,32−34^ The experience and developed knowledge regarding enrichment cultures and municipal activated sludge are based on similar selection principles for the enrichment of PHA-accumulating bacteria. Dynamic process environments with alternating presence and absence of carbon source, also known as “feast and famine”, have become standard practice for enrichment in this research community over 20 years.^16^ These selective environments exploit competitive advantage based on substrate uptake rate, which can favor PHA-accumulating bacteria because of the ability to quickly channel excess carbon in overflow metabolism.^35^ Nevertheless, feast–famine selective pressure does not necessarily enrich for superior PHA-accumulating bacteria in the absence of an intrinsic benefit to accumulate 0.9 rather than 0.6 gPHA/gVSS.^36^ The experience of the specific conditions that result in enrichment of *Plasticicumulans acidivorans* or similar species of bacteria suggests that extreme levels of PHA accumulation potential are not generic to survival. Those species that reach PHA content of 0.9 and not 0.6 gPHA/gVSS indicate that other factors govern the ability for super accumulators to dominate in certain feast–famine reactors and municipal WWTPs.^36,37^

### 4.2 Degree of Enrichment Determines the PHA Accumulation Potential in Municipal Activated Sludge

As observed in the present work, even if on average the PHA-storing phenotype in municipal activated sludge can accumulate up to 0.6 gPHA/gVSS, the observed PHA content levels for the municipal activated sludge were significantly lower, ranging from 0.18 to 0.42 gPHA/gVSS. WWTPs with higher biomass PHA content were shown to also exhibit a higher degree of enrichment. Selective pressures to enrich for the PHA-accumulating phenotype in municipal WWTPs are not sufficient to drive toward a high degree of enrichment for PHA-accumulating bacteria. Different factors may affect the biomass degree of enrichment. Factors include the influent wastewater quality as well as the WWTP bioprocess configuration with its conditions of operation.

The influent wastewater is normally composed of readily biodegradable soluble COD, e.g., volatile fatty acids, carbohydrates, or alcohols, and other forms of slowly biodegradable soluble and solid COD, e.g., proteins, humic acids, or cellulose. Bacteria can accumulate PHA using different kinds of readily biodegradable soluble COD. Nonetheless, volatile fatty acids are the preferred substrate for microbial PHA production. Other kinds of organic substrates will be directly linked to the growth of the non-PHA-accumulating bacteria.^38^ A higher volatile fatty acids fraction in the influent wastewater will be expected to result in improved selection in the WWTP.^39^ However, it has also been shown that influent municipal wastewater with low levels of VFAs in the readily biodegradable fraction of the influent will support significant selection pressure.^40^ Further insight is required on how the readily biodegradable fraction of municipal influent wastewater can be exploited to drive the biomass toward a higher degree of enrichment.

The WWTP process configuration may affect the degree of enrichment for PHA-accumulating bacteria: the feeding pattern of the influent wastewater, the presence or absence of a primary treatment, and the type of biological treatment process. How the influent wastewater is fed into the anaerobic, anoxic, or aerobic zones or to a selector or contact volume can influence development of the degree of enrichment.^41^ It has been reported that only a feast phase and not a famine period is strictly required for the enrichment of PHA-accumulating bacteria.^38^ In another recent pilot system study, a sequencing batch reactor under a feast and famine regime was used to treat municipal wastewater with low to negligible levels of volatile fatty acids. The pilot scale biomass performance for PHA production was compared to the full scale biomass. The implementation of a sequencing batch reactor enabled an idealized full-scale plug flow process with better feast conditions than the full-scale installation. This change in interpreted mixing and profile for concentrations for the pilot scale influent COD resulted in a significant increase in the maximum PHA content to 0.49 gPHA/gVSS compared to only 0.15 gPHA/gVSS for full scale activated sludge.^40^ This increase was assumed to be due to improved selection. If it is assumed that the PHA-accumulating fraction could accumulate an average of 0.6 gPHA/gVSS, from this work, an increase in the degree of enrichment from 0.12 to 0.64 volume-to-volume ratio is estimated. A deeper insight on selection pressure for municipal wastewater treatment activated sludge will require explicit coupling between configuration and operations with outcomes of the degree of enrichment methods applied in the present work.

Primary treatment is expected to lead to a higher degree of enrichment. Primary treatment can reduce the concentration of inert organic solids adsorbed in the activated sludge. Adsorbed inert solids effectively reduce the degree of enrichment of the solids. They will also hydrolyze and degrade more slowly in the process. This degradation may support growth of flanking populations of non-PHA storing microorganisms. Previously, a measurable impact of primary treatment on the maximum PHA content was not found.^15^ However, in the present study, WWTPs with primary treatment exhibited higher PHA content (0.40 gPHA/gVSS) and degree of enrichment (0.47 v/v) compared to WWTPs without a primary treatment (0.26 ± 0.06 gPHA/gVSS and 0.27 ± 0.07 v/v). These differences were statistically significant (*p* < 0.05).

The biological process configuration may influence both the degree of enrichment and biomass PHA content. WWTPs with either AO or A^2^O configurations may select for different microbial communities. In the present study, and in line with previous experience, WWTPs with AO configurations had a slightly higher biomass PHA content and degree of enrichment (0.33 ± 0.07 gPHA/gVSS, 0.36 ± 0.11 v/v) compared to WWTPs with A^2^O configurations (0.25 gPHA/gVSS, 0.29 v/v).^15^ However, the differences were not significant. Both configurations showed higher and lower biomass PHA contents. It may also be that the PHA accumulation method used in the present work is not the most suitable for polyphosphate-accumulating organisms. Polyphosphate-accumulating organisms are usually enriched under anaerobic/aerobic cycles and do not accumulate only PHAs but also polyphosphate and glycogen. For A^2^O WWTPs, it could be of interest to start the PHA accumulation under anaerobic conditions where PHA is produced and the polyphosphate and glycogen pool are depleted, followed by a subsequent aerobic phase, as proposed previously.^42^ Moreover, deepened comparative evaluations are required to understand what makes a given A^2^O (or AO) result in an activated sludge with higher or lower degrees of enrichment. Because both outcomes were observed, the configuration in itself was not a definitive determining factor in these cases.

Layered on top of the process configuration, operational conditions including temperature and solids retention time can affect the degree of enrichment for PHA-accumulating microorganisms. Temperature has been shown to be a factor for successful enrichment of PHA-accumulating bacteria in feast and famine reactors, especially at low solid retention times.^36,41,43^ Higher temperatures (ca. 30 °C) in enrichment reactors showed a consistent response toward polymer storage, while lower temperatures (ca. 20 °C) showed a mixed response of growth and storage. These results suggest a role of temperature in the competition between growth and polymer storage. Average annual temperatures for northern Europe are expected to be around 10 °C. Outcomes for degree of enrichment, with all other factors being similar, may be different for climates warmer than that of The Netherlands. The influence of solids retention time on selection for degree of enrichment has not been conclusive. Some research has reported that shorter solids retention times will result in higher PHA accumulation potentials.^39^ However, others have shown that solids retention times did not have a significant impact on the biomass PHA content.^44^

### 4.3 Strategies to Maximize PHA Production with Municipal Activated Sludge

It was found that a significant fraction of municipal activated sludge from a set of northern European WWTPs comprised PHA-accumulating bacteria. Independent of the source of the activated sludge, PHA-accumulators accumulated on average in the order of 0.6 gPHA/gVSS. However, the activated sludge degree of enrichment meant that the average biomass PHA contents were lower and in the range of 0.18–0.42 gPHA/gVSS. Methods to optimize the PHA production process with municipal activated sludge need to be considered. The following methods are proposed:

1 *Before the PHA accumulation process.* The degree of enrichment can be increased before the PHA accumulation, for instance, in the municipal WWTP, without the need to change the biological process by including a primary treatment or creating better feast conditions in the activated sludge process, as discussed above.
2 *In the PHA accumulation process.* The degree of enrichment may be increased directly in the PHA accumulation process if conditions for the selective growth of the PHA-accumulating biomass can be created. For Beverwijk WWTP activated sludge, PHA content and the fraction of PHA storing biomass steadily increased over the time of the accumulation without reaching a plateau level (Figure 2). This observation suggests that biomass growth was selective to the PHA-accumulating biomass fraction. Examples of simultaneous growth and PHA accumulation with enriched cultures can be found in the literature.^20,31,45^ However, these strategies have not been consistent in outcome, have resulted in low average PHA yields on the substrate, or involved a biomass with an already high degree of enrichment. Thus, greater insight is needed to define which conditions will promote consistently predictable concurrent selective growth and PHA accumulation during direct PHA accumulation using an activated sludge with lower starting degree of enrichment.
3 *After the PHA accumulation process.* The degree of enrichment may be increased if methods are implemented for the selective removal of non-PHA-containing biomass in the downstream process after the PHA accumulation. PHA and non-PHA biomass are expected to have different density. Disruption of floc structure will avail in principle a potential to separate PHA-rich fractions by gradient centrifugation.^46^ Direct accumulation of municipal activated sludge was found to result in clusters of the PHA-accumulating bacteria in most of the activated sludge samples. Similarly, the non-PHA biomass fraction may be selectively removed or digested. In pure culture PHA production, PHA-rich biomass has been digested by a species of mealworms resulting in fecal matter of high PHA purity.^47^ Similar experiments with PHA-rich biomass produced from activated sludge could be performed to test the feasibility of this approach.
